# Effect of red ginger powder (*Zingiber officinale* var. *rubrum*) as a feed additive for starter and finisher broiler chicken to increase immunoglobulin A and immunoglobulin Y expression and to prevent intestinal injury due to *Salmonella enteritidis* infection

**DOI:** 10.14202/vetworld.2022.1506-1514

**Published:** 2022-06-18

**Authors:** Herawati Herawati, Agri Kaltaria Anisa, Kurnianto Dwi Widiatmoko, Setiawan Surya Paku Alam, Islah Asyraf Diari, Zhella Happy Naprila, Rr. Lintan Ayu Kisya, Analita Puspabela, Fajar Shodiq Permata

**Affiliations:** 1 Department of Veterinary Public Health, Faculty of Veterinary Medicine, Universitas Brawijaya, Malang, 65151, Indonesia; 2 Department of Pharmacology, Faculty of Veterinary Medicine, Universitas Brawijaya, Malang, 65151, Indonesia; 3 Veterinary Science Undergraduate Program, Faculty of Veterinary Medicine Universitas Brawijaya, Malang, 65151, Indonesia; 4 Department of Histology, Faculty of Veterinary Medicine, Universitas Brawijaya, Malang, 65151, Indonesia

**Keywords:** bacterial isolation, fresh feces, natural immunomodulator, red ginger powder, *Salmonella enteritidis*

## Abstract

**Background and Aim:**

Salmonellosis is an infectious disease that often occurs in chickens and is caused by *Salmonella enteritidis*. The use of antibiotics to prevent this disease can result in the development of resistance in pathogenic bacteria, in addition to the presence of antibiotic residues in consumed carcasses. Red ginger (*Zingiber officinale* var. *rubrum*) has active compounds that potentially act as immunomodulators which increase specific and non-specific immune responses through the induction of cytokine production. This study was conducted to determine the effects of red ginger powder mixed in feed for starter and finisher broiler chickens, based on the evaluation of the expression of immunoglobulin A (IgA), histopathologic description of the ileum and cecum, IgA, and immunoglobulin Y (IgY) expression in the spleen, and the isolation count of *S. enteritidis* in fresh fecal samples.

**Materials and Methods:**

A total of 100 starter and 100 finisher Cobb broiler chickens were divided into four groups, designated as T0, T1, T2, and T3, respectively: Group T0 was fed commercial feed with no added 2% red ginger powder or *S. enteritidis* induction, and served as a negative control; Group T1 was inoculated with a 0.25 mL *S. enteritidis* oral induction (1 × 10^7^ colony-forming unit [CFU] [0.5 McFarland standard]), and served as a positive control; Group T2 was fed with feed containing 2% red ginger powder; while Group T3 was fed with feed containing 2% red ginger powder and was orally inoculated with *S. enteritidis* with a dose similar to T1. The normal feed was given on the 1^st^–7^th^ days. The mixture of 2% red ginger powder was given on the 7^th^–15^th^ days. The *S. enteritidis* was induced on the 15^th^ day (1 × 10^7^ CFU). Necropsy was performed on the 16^th^ day and tissues were fixed in 10% formalin and routinely processed for histopathologic and immunohistochemical analyses. The data were analyzed using a one-way analysis of variance test, Tukey’s analysis, and the Mann–Whitney *U* non-parametric statistical analysis test.

**Results:**

The 2% red ginger powder was found to significantly (p < 0.05) increase IgA expression and additionally decrease tissue damage in the cecum and ileum. It also increased IgA and IgY expression in the spleen. In addition, a decrease was observed in the *S. enteritidis* number isolated from finisher fresh feces, but none was found in the isolated starter fresh feces.

**Conclusion:**

These findings indicate that the addition of red ginger powder to chicken feed is a potential natural immunomodulator against *S. enteritidis* infection.

## Introduction

Salmonellosis is an infectious disease that attacks the digestive tract in poultry. Salmonella infection is also a major cause of foodborne disease in humans around the world. It is transmitted through contaminated water or food and often presents as acute gastroenteritis [1, 2]. *Salmonella enteritidis* is a pathogenic Gram-negative, non-spore-forming, short rod bacteria, and a member of the Enterobacteriaceae family [2]. *Salmonella* infections in humans primarily occur due to the consumption of contaminated chicken and poultry products [3]. The clinical manifestation of *S. enteritidis* varies in chickens, and can include weakness, depression, diarrhea, and mortality, especially in <1-week-old birds and those with immunosuppressive conditions [3, 4]. *S. enteritidis* is transmitted both vertically from eggs (transovarial), and horizontally from the environment [4]. *S. enteritidis* colonization and virulence are mediated by virulence plasmids and *Salmonella* Pathogenicity Island (SPI) genes. In the early stage of infection, SPI encode three secretion systems and translocate effectors across host cell membranes. This effector is essential for the bacterial invasion of host intestinal cells [5]. Subsequently, the bacteria localizes in the submucosa through specialized M-cells. This leads to intestinal inflammation characterized by heterophils, macrophages, erythrocytes, and immune cells which infiltrate into cecal luminal exudate and lamina propria [6]. This inflammation triggers the drainage of water into the gastrointestinal tract and causes diarrhea [5, 6]. Meanwhile, the bacteria also spread to other organs through body fluids, eventually infecting blood vessels, lymphatic tissue, the liver, spleen, and peripheral tissues [5, 7]. Bird eggs are colonized by *S. enteritidis* either in the yolk or albumin. It is deposited near the basement membrane of the highly vascularized theca wall before migrating through the perivitelline layer and invading ovarian granulosa cells [7].

Antibiotics have been the primary therapeutic agent used for both preventions of *Salmonella* infection and treatment. Antibiotics have been utilized prophylactically and in growth and health improvement strategies in poultry. However, this leads to antibiotic resistance which has rapidly increased within microbial communities [4, 8]. Antibiotic-resistant *Salmonella* have been isolated in most endemic areas of the world, notably in Southeast Asia and the Middle East [8]. Consequently, antibiotics are becoming less effective, and this development has encouraged researchers to develop novel alternatives to prevent and treat Salmonella infection [9].

Red ginger (*Zingiber officinale* var. *rubrum*) is already well known as a natural alternative in the medical field. It has phenolic acids, flavonoids, Vitamin C, curcumin, 6-gingerol, eugenol, and essential amino acids, along with antioxidant activity [10], and is reported to have antimicrobial, anti-inflammatory, immunomodulatory, and antioxidant effects, as well as free radical scavenging activity [11]. Innate immune system defenses activate macrophages and other phagocytes to recognize and kill the bacteria once they infect the host cell. These processes also result in the production of microbicidal substances, such as reactive oxygen species, nitric oxide, proteolytic enzymes, and lysozyme. These byproduct effects are counteracted by red ginger, which bioactively scavenges superoxide and hydroxyl radicals and also generates oxygen-containing free radicals. This immunomodulatory activity helps to prevent the intestinal mucosal structure from being damaged [12, 13]. Bioactive compounds in red ginger also stimulate mitogen-activated protein kinase to activate nuclear factor-kappa-β (NF-K*β*), using dynamic signaling which transmits bacteria stimuli information for a transcription factor which then stimulates cell proliferation and leukocyte differentiation [14]. In addition, the NF-kβ activation induces production of inflammatory factors such as cytokine interleukin (IL)-6, IL-8, and tumor necrosis factor-β [15, 16]. The production of these cytokines activates macrophages, enhances cytotoxicity, and mediates cellular immune response [17].

This study aims to highlight the applications of an alternative natural additive for chicken feed to achieve an immunomodulatory effect against *S. enteritidis* infection in broiler chickens.

## Materials and Methods

### Ethical approval

The study design was approved by the Animal Care and Use Committee, Brawijaya University, Malang, Indonesia (No: 1147-KEP-UB). All experiments were carried out following the Ministry of Health Indonesia, International Guiding Principles for Biomedical Research Involving Animal, and associated guidelines based on animal welfare principles.

### Study period and location

The study was conducted from August to December 2019. The chicken rearing and feeding were done at Malang Agricultural Development Polytechnic, the tissue processing was done at Histology Laboratory of Veterinary Medicine Brawijaya University, and the immunohistochemistry preparation was done at Biology Molecular Laboratory of Medicine Faculty Universitas Brawijaya.

### Experimental animals

A total of 100 starters and 100 finisher Cobb broiler chickens were divided into four groups with five repetitions. Each repetition consisted of five chickens, respectively. The starter chickens were 16 days old with an average body weight of 100–400 g, and the finisher chicken were 35 days old and weighed an average of 1.5–2 kg. The chickens were fed with normal feed pellets BR511 and CP511, at 10 g/chicken/day, and without 2% red ginger powder addition or *S. enteritidis* induction for the first 7 days (1^st^–7^th^ days). The 2% red ginger powder was added to the feed for the next 8 days (8^th^–15^th^ days). Then, the *S. enteritidis* was induced on the 15^th^ day (1 × 10^7^ colony-forming unit [CFU]/mL). Finally, a necropsy was performed on the 16^th^ day. The cecum, ileum, and spleen were stored in 10% formalin and collected together with the fresh feces samples. They were then inserted into a labeled-plastic zipper bag and stored at 4°C. The histopathology and immunohistochemistry slides were then prepared and analyzed.

### *S. enteritidis* preparation

The *S. enteritidis* used in the study was obtained from the Veterinary Center, Wates, Yogyakarta, Indonesia. Pure *S. enteritidis* bacteria isolates were stored in nutrient agar at 4°C, and then subcultured in *Salmonella* *Shigella* Agar (SSA) and harvested. The colonies were suspended in 100 mL of distilled water, homogenized, and equalized with 0.5 McFarland standard (bacteria standard; 10^8^ CFU/mL), which had been diluted to a concentration of 10^7^ CFU/mL (1:10).

### Feces sample preparation and isolation

Feces sampling was conducted on the 16^th^ day for the starter phase and on the 35^th^ day for the finisher phase, 24 h after *S. enteritidis* induction (1 × 10^7^ CFU/mL). Feces were taken randomly per repetition for each group. The fresh feces samples were collected in the afternoon. Fresh feces were considered suitable for *Salmonella* spp. isolation and identification when a large amount of it was found in the lower gastrointestinal tract [18]. Approximately 0.1 g of feces samples were homogenized with 0.9 mL phosphate-buffered saline (PBS) (1:9) using a vortex, then cultured in SSA using the streak plate method. Eventually, the media were incubated at 37°C for 24–48 h. Colony calculations were carried out using a colony counter, with a formula as follows:


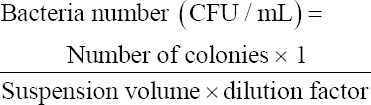


### Histopathology and immunohistochemistry preparation

The organ samples were fixed in 10% formalin for 24 h and then trimmed to 1 × 1 × 0.5 cm and inserted into tissue cassettes. The tissue cassettes were immersed into 70%, 80%, 90%, and 95% ethanol and absolute ethanol I, II, and III gradually for 1 h, respectively, then immersed into xylene I, II, and III for 10 min, respectively. Next, the organs were embedded in paraffin blocks and set. Subsequently, the blocks were sectioned at 5 mm thick samples and placed onto a cover glass. They were then stained using hematoxylin-eosin staining and prepared for immunohistochemical analysis.

The hematoxylin-eosin staining process was started with the immersion of the section placed on the glass objects in xylene I, II, and III for 20 min, continued with absolute ethanol III, II, and III, then graded ethanol 95%, 90%, 80%, and 70%, for 5 min each, respectively. Afterward, the slides were immersed in hematoxylin solution for 15 min, soaked in acidic ethanol for 4 s, washed with running water for 20 min, and immersed in eosin for 20 min. The slides were then immersed in graded ethanol 70%, 80%, and 90%, absolute ethanol I, II, and III for 4 s, respectively, followed by immersion in xylene I, II, and III for 10 min each. Eventually, the slides were mounted with Entellan, covered with a cover glass, and observed using a light microscope.

The first step in the immunohistochemistry preparation process was the placement of the sample on the glass objects, which were then immersed in xylene I and II for 5 min, continued to absolute ethanol I, and then graded ethanol 90%, 80%, and 70%, for 5 min, respectively. The slides were then flooded with distilled water for 5 min and dripped with cell staining buffer for 10 min. It was next blocked with peroxidase for 40 min, then washed with PBS 3 times. The area around the sample was dried and cleaned carefully and dripped with immunoglobulin A (IgA) antibodies (cat. LS-C210723, LS Bio, USA, for spleen, caecum, ileum, 1:100) or immunoglobulin Y (IgY) antibodies (cat. LS-C56695, LS Biol, USA, for spleen, 1:100), diluted with FBS. The slides were then incubated in a temperature room with dark container or blackout area for 1 hour and washed in PBS 3 times. The slides were next dripped with diamono benzidine for 20 min and then rinsed with distilled water. Subsequently, the slides were dripped with Mayer’s Hematoxylin, and then dripped with pH 8+ water, rinsed with distilled water, and dried. Eventually, the slides were mounted with entellan and covered with a cover glass and observed using a light microscope.

### Statistical analysis

The histopathology results were observed and analyzed descriptively using a light microscope. Immunohistochemistry results were analyzed using the ImageJ (National Institutes of Health, USA) ImmunoRatio plugin. The quantitative data were processed with a one-way analysis of variance and further analyzed using Tukey’s test. Meanwhile, the *S. enteritidis* count results were analyzed with the Mann–Whitney *U* test using the IBM Statistical Package for the Social Science Statistics version 25 (IBM, Armonk, NY, USA).

## Results

### Histopathology of starter chicken ileum and cecum

**Figure-1:**
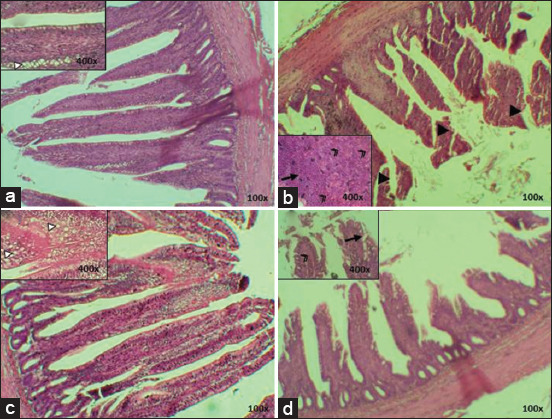
Histopathology starter ileum; (a) T0; (b) T1; (c) T2; (d) T3, (►) villi rupture, (∆) goblet cell hyperplasia, (→) inflammatory cell infiltration, (“) hemorrhage (HE) 100× and 400×.

The first treatment group (T0) showed no damage to the villi, which was indicated by the intact-shaped vili of the ileum . The second treatment group (T1) showed villi damage, hemorrhage, and inflammation. The third treatment group (T2) showed no damage on villi surfaces, where the villi were well-arranged in height and even width. Goblet cell hyperplasia was also seen. The fourth treatment group (T3) showed mild damage and shortened villi (Figure-1).

**Figure-2:**
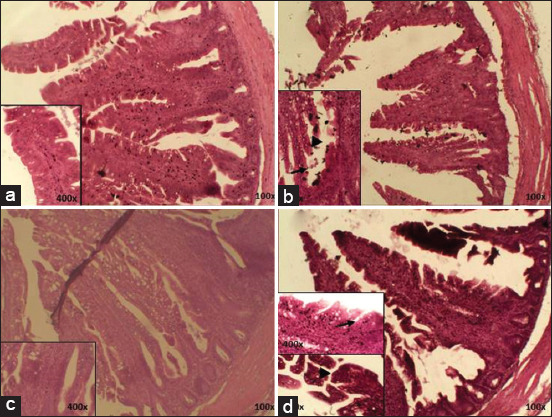
Histopathology of starter cecum; (a) T0; (b) T1; (c) T2; (d) T3, (►) villi rupture, (→) epithelium erosion (HE) 100× and 400×.

The cecum of T0 starter chicken appeared normal, whereas the simple columnar epithelium appeared to be normal. Meanwhile, the T1 group showed a different structure of the cecum mucosa with epithelial erosion and villi rupture. Analysis of the results of T2 showed no damage and the structures were similar to those of the negative control (T0), with no epithelial erosion or villi rupture. The T3 treatment group showed minimalized damage, which was better than seen in the positive control (T1) (Figure-2).

### Histopathology of finisher chicken ileum and cecum

**Figure-3:**
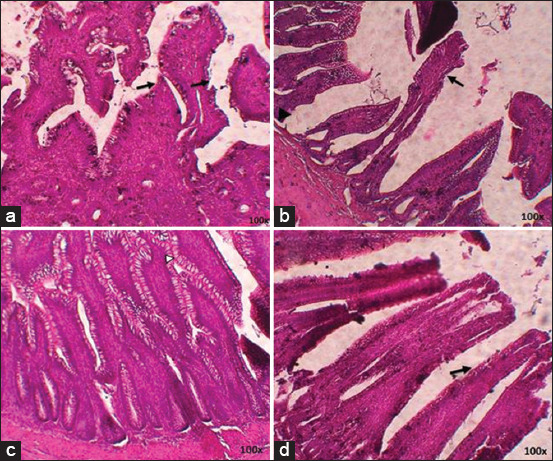
Histopathology finisher ileum; (a) T0; (b) T1; (c) T2; (d) T3, (►) villi rupture, (→) epithelium erosion (HE), (∆) goblet cell (HE) 100× and 400×.

The T0 group showed good density and normal structure of the ileum. The positive control (T1) showed villi rupture, characterized by damaged Lieberkuhn crypts along with low structure density. In addition, the T2 showed no epithelium erosion with goblet cells. In the T3 group, tunica mucosa erosion was seen, but the villi were observed to have good cell density (Figure-3).

**Figure-4:**
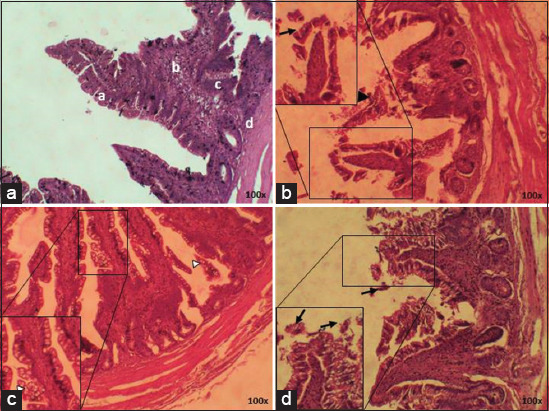
Histopathology finisher cecum; (a) T0; (b) T1; (c) T2; (d) T3, a. epithelium, b. lamina propria, c. tunica mucosa, d. tunica muscularis (►) villi rupture, (→) epithelium erosion (HE), (∆) goblet cell hyperplasia (HE) 100× and 400×.

The cecum showed a normal structure, characterized by parts of the structure which could be observed, including cell density in the T0 group. The T2 group showed goblet cell hyperplasia, while the T1 and T3 groups showed villi rupture, with T3 showed more mild damage than T1 (Figure-4).

### IgA expression of starter chicken ileum and cecum

**Figure-5:**
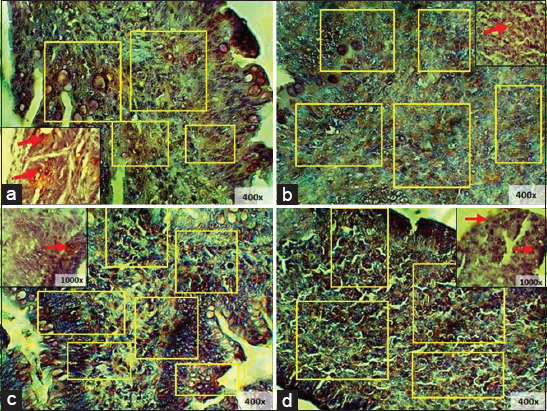
Immunohistochemistry of starter’s ileum: (a) T0; (b) T1; (c) T2; (d) T3, (□) immunoglobulin A expression 400× and 1000×.

The IgA expression of starter chicken ileum showed the highest levels in T2 and the lowest levels in T1 (Figure-5). Statistical analysis showed a significant difference in IgA expression in the ileum with the addition of red ginger powder (p < 0.05). This finding was further subjected to Tukey’s test, where T0 was found to show a significantly different average of IgA expression area (p < 0.05) compared to T1, but T0 was not significantly different (p > 0.05) from T2 or T3 (Table-1).

**Table 1:** IgA expression in starter chicken ileum.

Treatment group	IgA expression area (%)
T0 (negative control)	32.36 ± 7.55^b^
T1 (positive control)	27.02 ± 5.25^a^
T2 (normal feed+2% red ginger powder)	36.91 ± 11.39^a,b^
T3 (normal feed+2% red ginger powder+S*almonella enteritidis*)	34.39 ± 7.49^b^

*Different notations indicate a significant difference between treatments (p < 0.05). IgA = Immunoglobulin A

**Figure-6:**
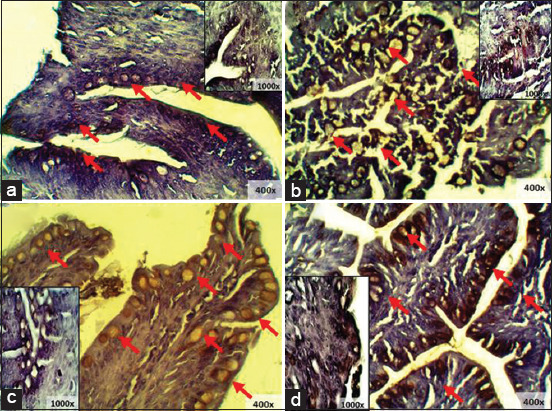
Immunohistochemistry of starter’s cecum: (a) T0; (b) T1; (c) T2; (d) T3, (→) immunoglobulin A expression 400× and 1000×.

The IgA expression of starter chicken cecum showed that T2 had a higher IgA expression compared to T0, while T3 showed a higher expression than T1 (Figure-6). Statistical analysis showed significant differences in IgA expression in the cecum with the addition of red ginger powder (p < 0.05). The result was then subjected to Tukey’s test; and the IgA production was found to be lower compared to T1, while T2 and T3 showed greater improvement compared to the control groups (Table-2).

**Table 2:** IgA expression in starter chicken cecum.

Treatment group	IgA expression area (%)
T0 (negative control)	15.28 ± 11.15^a^
T1 (positive control)	10.07 ± 9.0^a^
T2 (normal feed+2% red ginger powder)	37.96 ± 27.77^b^
T3 (normal feed+2% red ginger powder+S*almonella enteritidis*)	17.61 ± 2.81^a^

*Different notations indicate a significant difference between treatments (p < 0.05). IgA = Immunoglobulin A

### IgA expression of finisher chicken ileum and cecum

**Figure-7:**
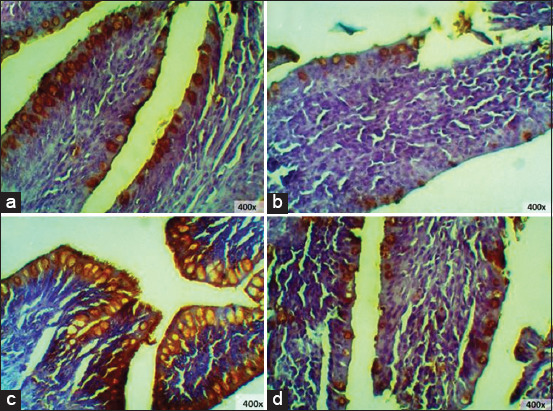
Immunohistochemistry of finisher’s ileum: (a) T0; (b) T1; (c) T2; (d) T3, (□) immunoglobulin A expression 400×.

IgA expression of finisher chicken ileum showed that T0 and T3 had nearly the same expression levels. Meanwhile, the other treatment groups showed significant differences (Figure-7). Statistical analysis showed significant differences with the addition of red ginger powder to IgA expression in the cecum (p < 0.05). This result was then further subjected to Tukey’s test, and it was found that T2 IgA expression significantly increased compared to all other treatment groups and that T1 was significantly lowest in comparison to the other treatment groups (Table-3).

**Table 3:** IgA expression in finisher chicken ileum.

Treatment group	IgA expression area (%)
T0 (negative control)	14.69 ± 3.63^b^
T1 (positive control)	8.62 ± 2.03^a^
T2 (normal feed+2% red ginger powder)	19.24 ± 4.00^c^
T3 (normal feed+2% red ginger powder+S*almonella enteritidis*)	12.71 ± 2.24^b^

*Different notations indicate a significant difference between treatments (p < 0.05). IgA = Immunoglobulin A

**Figure-8:**
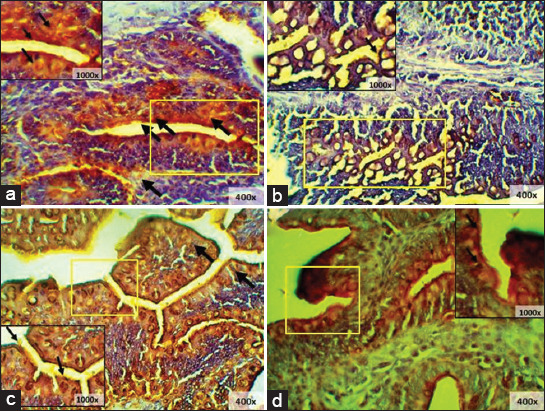
Immunohistochemistry of finisher’s cecum: (a) T0; (b) T1; (c) T2; (d) T3, (□) immunoglobulin A expression 400× and 1000×.

The IgA expression of finisher chicken cecum revealed the highest IgA expression in group T2, while T1 showed the lowest expression of IgA (Figure-8). Statistical analysis showed that the addition of ginger powder induced significant differences in IgA expression in the cecum (p < 0.05). The result was then further subjected to Tukey’s test, and T1 was found to have the highest IgA levels compared to the other treatment groups (Table-4).

**Table 4:** IgA expression in finisher chicken cecum.

Treatment group	IgA expression area (%)
T0 (negative control)	51.28 ± 29.78^a^
T1 (positive control)	30.48 ± 28.17^b^
T2 (normal feed+2% red ginger powder)	55.36 ± 28.91^a^
T3 (normal feed+2% red ginger powder+S*almonella enteritidis*)	54.76 ± 27.27^a^

*Different notations indicate a significant difference between treatments (p < 0.05). IgA = Immunoglobulin A

### IgA and IgY expression of finisher spleen

**Figure-9:**
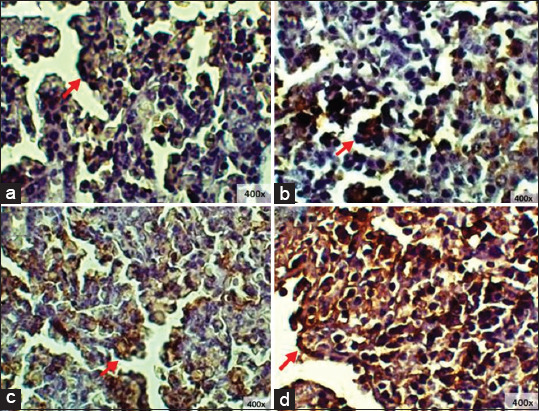
Immunohistochemistry of finisher’s spleen without *S. enteritidis* infection: (a) T0 immunoglobulin A (IgA) expression; (b) T2 IgA expression; (c) T0 immunoglobulin Y (IgY) expression; (d) T2 IgY expression, (→) Ig expression.

**Figure-10:**
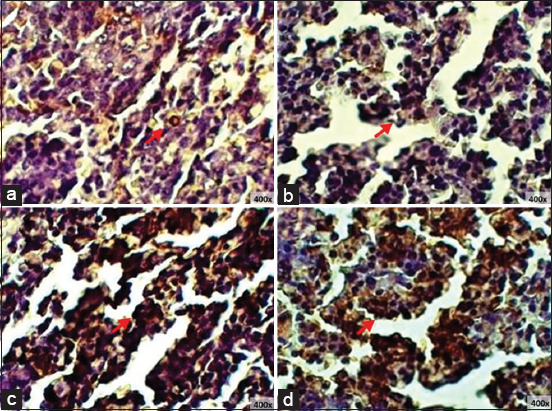
Immunohistochemistry of finisher’s ileum with *S. enteritidis* infection: (a) T1 immunoglobulin A (IgA) expression; (b) T3 IgA expression; (c) T1 immunoglobulin Y (IgY) expression; (d) T3 IgY expression, (→) IgA expression.

Comparison between the treatment groups without *S. enteritidis* infection (T0 and T2) showed that T2 had the highest level of IgA and IgY expression (Table-5). This was reflected by the microscopy results, where the brown-colored areas were more dominant in T2 compared to T0 (Figure-9). Meanwhile, a comparison between the groups treated with *S. enteritidis* infection (T1 and T3) showed that the T3 IgA expression was higher than that of T1; this indicates that red ginger powder influenced a higher IgA expression in the spleen. In contrast, there was no significant difference in IgY expression between T3 and T1 (Table-6), as confirmed by microscopy (Figure-10).

**Table 5:** IgA and IgY expression in finisher chicken spleen without *Salmonella enteritidis* induction.

Treatment group	IgA expression area (%)	IgY expression area (%)
T0 (negative control)	19.22 ± 6.28	29.03 ± 12.43
T2 (normal feed+2% red ginger powder)	22.25 ± 5.87	46.96 ± 8.82

IgA = Immunoglobulin A, IgY = Immunoglobulin Y

**Table 6:** IgA and IgY expression in finisher chicken spleen with *S. enteritidis* induction.

Treatment group	IgA expression area (%)	IgY expression area (%)
T1 (positive control)	14.82 ± 5.15	35.89 ± 10.44
T3 (normal feed+2% red ginger powder+S*almonella enteritidis*)	16.22 ± 5.23	35.14 ± 9.35

IgA = Immunoglobulin A, IgY = Immunoglobulin Y

### *S. enteritidis* number from fresh feces isolation count

There were no *S. enteritidis* colonies obtained from any starter chickens from any of the treatment groups (Table-7). In contrast, the colonies presented in T1 and T3 of the finisher chicken treatment groups are shown in Table-8. The colonies which appeared were 22 × 10^−1^ CFU/mL for T1, and 14 × 10^−1^ CFU/mL for T3. However, there were no significant differences observed among the colonies of the finisher treatment groups (Table-9).

**Table 7:** Fresh feces isolation results in starter chicken on *Salmonella Shigella Agar* medium.

No.	Treatment group	Sample	Colony number (×10^−1^ CFU/mL)	Mean (×10^−1^ CFU/mL)
1	Negative control	P1U1	0	0
2		P1U2	0	
3		P1U3	0	
4		P1U4	0	
5		P1U5	0	
6	Positive control	P2U1	0	0
7		P2U2	0	
8		P2U3	0	
9		P2U4	0	
10		P2U5	0	
11	Normal feed + 2% red ginger powder	P3U1	0	0
12		P3U2	0	
13		P3U3	0	
14		P3U4	0	
15		P3U5	0	
16	Normal feed+2% red ginger powder + S*almonella enteritidis*	P4U1	0	0
17		P4U2	0	
18		P4U3	0	
19		P4U4	0	
20		P4U5	0	

CFU = Colony forming unit

**Table 8:** Fresh feces isolation results in finisher chicken on *Salmonella Shigella Agar* medium.

No.	Treatment group	Sample	Colony number (×10^−1^ CFU/mL)	Mean (×10^−1^ CFU/mL)
1	Negative control	P1U1	0	0
2		P1U2	0	
3		P1U3	0	
4		P1U4	0	
5		P1U5	0	
6	Positive control	P2U1	49	22
7		P2U2	5	
8		P2U3	10	
9		P2U4	5	
10		P2U5	39	
11	Normal feed + 2% red ginger powder + S*almonella enteritidis*	P3U1	29	14
12		P3U2	0	
13		P3U3	8	
14		P3U4	19	
15		P3U5	13	
16	Normal feed + 2% red ginger powder	P4U1	0	0
17		P4U2	0	
18		P4U3	0	
19		P4U4	0	
20		P4U5	0	

CFU = Colony forming unit

**Table 9:** Mann–Whitney Non-parametric statistical test result.

Sample collection	T0 and T1	T1 and T3	T3 and T2	T1 and T2
16^th^ day-starter	**-**	**-**	**-**	**-**
35^th^ day-finisher	0.027	0.753*	0.113*	0.014

* Significantly has no differences ( > 0.05)

## Discussion

Red ginger feed addition in the T3 treatment groups was compared to the positive control groups. Red ginger seemed to work by an activation path which was the result of its bioactive compounds, which helped to protect the intestines from infection. It is known to contain phenolic acids, flavonoids, Vitamin C, curcumin, 6-gingerol, eugenol, and essential amino acids, along with having antioxidant activity [10]. Pathogenic bacteria that infect host bodies attach to the surface of the intestinal villi, using it as a growth medium which eventually causes tissues to rupture. The mucosal barrier sites coordinate the cellular response in homeostasis and epithelial cell balance to line the incoming bacteria. The goblet cells provided mucus, a carbohydrate-rich, gel-like substance that prevents penetration into underlying intestinal tissue [19]. The histological findings for chickens fed red ginger revealed better ileum and cecum structure which indicated normal absorption of nutrition in the digestive process. In modulated intestinal mucosa, the goblet cells first inhibit the bacteria by secreting mucus. In cases when this first barrier cannot stop the invasion, immunomodulators will help the cellular defensive systems of the host’s body by activating lymphocytes and macrophages. These then suppress the pathogenic bacteria population in the chicken gut. The structural damage can be worse if the chicken feed is not balanced with natural active compounds, which can suppress the pathogenic bacteria. In addition, pathogenic bacteria cause ruptures of the infected intestinal organs. As the histopathological changes were found, villi erosion thins out intestinal epithelium, accompanied by villi ruptures that showed loss of epithelium to the mucosa layer. It is reported that *S. enteritidis* infection causes desquamation of mucosal epithelium and denatured villi, resulting in necrotic cell mass accumulation in the intestinal lumen. In addition, severe inflammatory cell infiltration and intestinal gland changes appeared in histological analyses [20]. The more defective invasion can increase the structural damage and can decrease nutrition absorption levels and feed intake, thereby resulting in sub-optimal chicken growth.

In this study, the area of IgA expression in the positive control groups showed a decrease in IgA expression compared to the other groups. A decrease in IgA indicates a specific immunity activation against the *S. enteritidis* infection [21]. Mucosal lymphoid produces IgA, which plays a role in preventing bacterial invasion through humoral immunity, and the specific IgA antibody prevents pathogenic bacteria from adsorbing and entering epithelial cells. When Thelper2 is regulated, the primary cytokines secreted include IL-2, IL-4, and IL-10, which induce B cell proliferation, resulting in IgA and IgY enhancement [17, 22]. IgY is also known as immunoglobulin yolk, which is present in egg yolk and a major circulating antibody in chicken. IgY plays a similar biological role as IgG in mammals [23]. IgY has been reported to be resistant to intestinal protease indigestion [23, 24]. The chickens fed with red ginger powder in the *S. enteritidis* treatment group showed better structural repair than the positive control. This result is related to IgY immune activity in toxin neutralization, bacteria agglutination, and adhesion inhibition of bacteria. Inhibition of bacteria adhesion mechanism is considered the primary action of IgY in the prevention of diarrhea [23], and the IgA function in preventing bacteria from adsorbing and entering epithelial cells [22]. So suggests that the invasion is not penetrating the underlying structure of intestinal mucosa and submucosa; thereby resulting in a better histological structure resulting from the addition of red ginger compared with the positive control group.

No colonies of *S. enteritidis* were found in the starter phase. This could have occurred because the *S. enteritidis* had not entered its incubation period or shed cecal content within 24 h post-induction. Colonization and fecal shedding of *S. enteritidis* through experimental oral infection of chicks or hens transpire for several months [25, 26], and can persist for extended periods without showing symptoms [27]. The absence of colonies could also be due to host defenses, primarily in the intestinal tract. The mucus layer, secreted by goblet cells, covers epithelial cells and forms an integral structural component that naturally protects intestinal epithelium from damage caused by food and digestive secretions. It is renewed continuously to prevent bacteria from persisting on the epithelial surface and acts as a trap to block their access to the inner structure of the intestine [28]. The addition of red ginger powder showed a decreasing count (14 × 10^−1^ CFU/mL), compared to the positive control (22 × 10^−1^ CFU/mL) on average. These results prove that red ginger increases immunity and it has antibacterial properties against *S. enteritidis* infection.

## Conclusion

Based on the results obtained, it can be concluded that a 2% red ginger powder addition in broiler and finisher chicken feed may reduce the levels of organ damage resulting from *S. enteritidis* infection. The active compound in red ginger can act as immunomodulators by enhancing IgA and IgY expression, as well as lowering *S. enteritidis* fresh fecal isolation numbers in finisher chicken.

## Authors’ Contributions

HH: Research implementation. AKA: Preparation of bacteria induction and controlling red ginger feeding. KDW: Analyzed IgA and IgY expression of the spleen in starter phase and finisher phase. SSPA: Analyzed histopathology of ileum and IgA expression in the finisher phase. IAD: Analyzed histopathology of the caecum and IgA expression in the finisher phase. ZHN: Analyzed histopathology of the ileum and IgA expression in the starter phase. RLAK: Analyzed histopathology of the caecum and Ig expression in the starter phase. AP: Counted fecal bacteria in the starter phase and finisher phase of chicken. FSP: Conducted immunohisto­chemistry stain and statistical analysis. All authors have read and approved the final manuscript.
